# Case-Control Study to Assess the Association between Epilepsy and *Toxocara* Infection/Exposure

**DOI:** 10.3390/microorganisms9102091

**Published:** 2021-10-03

**Authors:** Ali Alizadeh Khatir, Mahdi Sepidarkish, Mohammad Reza Rajabalizadeh, Solmaz Alizadeh Moghaddam, Saeed Aghapour, Saeed Mehravar, Peter J. Hotez, Robin B. Gasser, Ali Rostami

**Affiliations:** 1 Mobility Impairment Research Center, Health Research Institute, Babol University of Medical Sciences, Babol 4714871167, Iran; alizade.ali83@yahoo.com; 2 Department of Biostatistics and Epidemiology, School of Public Health, Babol University of Medical Sciences, Babol 4714871167, Iran; mahdi.sepidarkish@gmail.com; 3 Student Research Committee, Babol University of Medical Sciences, Babol 4714871167, Iran; m.rajabalizadeh1374@gmail.com (M.R.R.); alizade.soli@gmail.com (S.A.M.); 4 Department of Neurosurgery, Faculty of Medicine, Mazandaran University of Medical Sciences, Sari 1353447416, Iran; aghapour.saeed@yahoo.com; 5 Department of Epidemiology and Statistics, School of Public Health, Tehran University of Medical Science, Tehran 1666663111, Iran; saeed.mehravar@gmail.com; 6 Texas Children’s Hospital Center for Vaccine Development, Department of Pediatrics and Molecular Virology and Microbiology, National School of Tropical Medicine, Baylor College of Medicine, Houston, TX 77030, USA; hotez@bcm.edu; 7 Department of Veterinary Biosciences, Melbourne Veterinary School, The University of Melbourne, Parkville, VIC 3010, Australia; 8 Infectious Diseases and Tropical Medicine Research Center, Health Research Institute, Babol University of Medical Sciences, Babol 4714871167, Iran

**Keywords:** *Toxocara*, epilepsy, association, case-control study

## Abstract

Although causes and etiology of epilepsy are mostly obscure, some zoonotic parasites, such as *Toxocara* species, have been proposed as a risk factor for this disease. Here, we conducted an age-matched case-control study to evaluate whether there is an association between epilepsy and the presence of serum antibodies to *Toxocara* in incident cases. We included 94 idiopathic epileptic patients as cases, and—from the same geographical region—88 people with no own history of epilepsy or neurological disease as control subjects. Epilepsy was confirmed by a physician using the International League Against Epilepsy (ILAE) definition. All participants were screened for the anti-*Toxocara* IgG serum antibody by enzyme-linked immunosorbent assay (ELISA). Univariate and mutltivariate statistical analyses were applied to calculate the crude and adjusted odds ratios (OR) and 95% confidence intervals (CIs). Anti-*Toxocara* serum antibody was detected in 37 epileptic patients and in 23 control subjects, giving respective seroprevalences of 39.3% (95% CI, 29.4–49.9%) and 26.1% (95% CI, 17.3–36.5%), respectively. Adjusted multivariate logistic regression analysis estimated an OR of 2.38 (95% CI, 1.25–4.63), indicating a significant association between epilepsy and *Toxocara* seropositivity. There was also a significant association between seropositivity to *Toxocara* and partial (OR, 2.60; 95% CI, 1.14–6.04) or generalized (OR, 2.17; 95% CI, 1.09–4.40%) seizures. Findings from the present study of incident epileptic cases support previous studies proposing that *Toxocara* infection/exposure is a risk factor for epilepsy. However, further well-designed population-based surveys and mechanistic/experimental studies in animal models are required to better understand the reason(s) for this association.

## 1. Introduction

Epilepsy is a serious neurological condition with a worldwide distribution, and characterized by abnormal brain activity that causes seizures and/or other neurological, cognitive, and psychological problems [1]. The burden of epilepsy is high; it is reported that at least 7 of every 1000 persons have epilepsy during their life-time, which means that ~50 million people of all ages are affected worldwide, relating to 13 million disability-adjusted life years (DALYs) and 0.5% of the global burden of disease (GBD) [2]. In spite of these figures, epilepsy is often neglected in public health agendas. There is a higher incidence of epilepsy in low- and middle-income countries (LMICs, 139 per 100,000 person-years) than in high-income countries (30–50 per 100,000 person-years) [2]. Current evidence indicates that 80% of people with epilepsy live in LMICs, most of whom do not have access to effective chemotherapy or clinical management [2]. While the specific etiology of epilepsy is not established for most patients (~60%), this disease is recognized as being multi-factorial, involving both genetic and environmental components [3]. The World Health Organization (WHO) indicates that 25% of epilepsy cases are preventable, such that there is an urgent need to identify risk factors for this disease [4]. Key, modifiable risk factors include infections, stroke, perinatal insults and traumatic injuries of the brain [5]. Neurotropic parasites including *Toxoplasma gondii* (causing toxoplasmosis) *Plasmodium falciparum* (malaria), *Taenia solium* (neurocysticercosis), *Onchocerca volvulus* (onchocerciasis) and *Toxocara* species (toxocariasis) can be important causative agents of infections of the central nervous system (CNS) [5]. The links between onchocerciasis and nodding syndrome and other forms of epilepsy are only now emerging [6].

*Toxocara canis* and *T. cati* are particularly significant zoonotic nematodes of canids and felids, respectively [7]. Humans are accidental hosts and become infected mainly through the accidental ingestion of eggs of *Toxocara* from contaminated soil, food or water and sometimes via eating undercooked or raw meat carrying *Toxocara* larvae [7]. Human *Toxocara* infection and toxocariasis (the disease) are important worldwide, and current estimates indicate that >1.4 billion people have been exposed to, or are infected with, *Toxocara* species [8]. In Iran, estimates indicate that ~6 (mean; range: 3–10%) of people have specific serum antibodies against *Toxocara* [8]. Moreover, ~16% and 26% of dogs and cats, respectively, are infected with *Toxocara*, and 20% of public places are contaminated with *Toxocara* eggs [7]. 

Toxocariasis of humans manifests itself in four clinical forms: visceral larva migrans (VLM), ocular larva migrans (OLM), covert/common toxocariasis (CT) and neurotoxocariasis (NT) [9]. NT is caused by the migration of larvae in the CNS, and is reported to occur commonly in middle-aged males (sex-ratio: 1.49; median age: 42 years) and less so in children of <18 years of age [10]. NT is characterized by encephalitis, meningitis, myelitis and/or cerebral vasculitis, but asymptomatic infection is common [10,11]. There are also proposed links between NT and developmental delays and cognitive impairments, especially in children [12]. MRI findings of neurotoxocariasis include subcortical, cortical or deep white-matter lesions with variable enhancement, which can associate with hydrocephalus, leptomeningeal or spinal cord involvement [13]. Findings from a range of epidemiological studies and meta-analyses [11,14,15,16,17,18,19] have indicated that *Toxocara* infection/exposure might play an under-appreciated role in inducing or contributing to neurological disorders, such as multiple sclerosis, schizophrenia, Parkinson’s, Alzheimer’s and/or epilepsy. 

Based on GBD 2016 estimates, there were 344,959 cases of epilepsy in Iran associated with 153,434 DALYs, and 765 people died from epilepsy and/or associated disorders [2]. Due to the high burden and public health importance of epilepsy in Iran, there is a need to explore its potential links with toxocariasis due to its high prevalence in Iran, as well as other LMICs [20,21,22,23,24,25]. Although numerous clinical studies of human epilepsy have been conducted in Iran and worldwide, all of them recruited prevalent epileptic patients as the case-group; thus, it was not possible to exclude a reverse causality, in which the development of epilepsy acted as a risk factor for *Toxocara* infection [17]. Here, we follow the recommendation from a recent meta-analysis [17] to attempt to better understand the causality of epilepsy, and conduct a matched case-control study of incident epileptic patients versus matched control subjects.

## 2. Materials and Methods

### 2.1. Study Site

This study was conducted in the Rouhani Hospital, a referral hospital in Babol, Mazandaran province, in northern Iran, between January 2019 and September 2020. Most people in this region are involved in farming animals and rice. The climate in the Mazandaran province is hot-humid in summer and mild-humid in winter, with an average annual temperature of ~18 °C, high annual precipitation (>800 mm) and high relative humidity (>70%), which provides favorable conditions for the transmission of many parasites, including *Toxocara* spp. [26]. A previous epidemiological study demonstrated that > 23.5% of people in this region have specific anti-*Toxocara* IgG serum antibodies, indicating infection with, or exposure to, species of *Toxocara* [26].

### 2.2. Study Population and Design

This human case-control study was approved by the Research Ethics Committee of the Babol University of Medical Science, Babol, Iran (permit no. IR.MUBABOL.HRI. REC.1399.105). All participants provided informed consent, and a structured questionnaire was used to obtain sociodemographic data. Clinical and neurological data/information were recorded and critically examined by an expert neurologist (A.A.K.). The incident cases were 94 patients diagnosed with ‘idiopathic epilepsy’, according to the International League Against Epilepsy (ILAE) guidelines [27]. Individuals were considered as ‘epileptics’ if they had at least two unprovoked seizures within a period of >24 h, and if the cause of their epilepsy was unknown; people with febrile convulsions, seizures caused by alcohol, intoxication by drugs or other substances, eclampsia or any other neuropathological condition were excluded. According to the ILAE classification and using electroencephalogram (EEG) records and clinical data, all patients with epilepsy (PWE) were stratified according to whether they had focal or generalized seizures; patients whose epilepsy could not be classified were excluded. Moreover, PWE who were mentally retarded and those with abnormal neurological findings, detected by computed tomography (CT) scans or magnetic resonance imaging (MRI), were also excluded. The group of control subjects were 88 age-matched (±3 years), healthy individuals referred to the General Health Outpatient Clinic (Rouhani Hospital); none of these subjects had a history of epilepsy or any other neurological disease (confirmed by a neurologist), although some of them indicated that they had relatives or parents with a history of epilepsy (Table 1).

### 2.3. Sample Collection and Laboratory Analysis

Blood samples were collected from individual participants (*n* = 182) and immediately centrifuged at 1000× *g* for 5 min, serum collected, aliquoted and frozen at −20 °C. Specific anti-*Toxocara* IgG serum antibodies were detected using a commercial enzyme linked immunosorbent assay (ELISA) kit with a diagnostic sensitivity and specificity of >95% (NovaTec Immunodiagnostics, Dietzenbach, Germany). The test results were recorded as international units (IU), as recommended. According to the instructions, respective values of <9.0 IU/mL, 9.0–11.0 IU/mL and >11.0 IU/mL were recorded as ‘test-negative’, ‘suspicious’ and ‘test-positive’ for anti-*Toxocara* IgG serum antibodies. Laboratory testing was conducted in a blinded manner, so that the expert technician who tested the samples was not aware of the health status of individuals whose serum samples were tested.

### 2.4. Statistical Analysis

All analyses were done by Stata statistical software (v.16 Stata Corp., College Station, TX, USA). The seroprevalence, based on anti-*Toxocara* serum antibodies in individual epileptic patients or healthy controls, was given as a relative percentage with a binomial 95% confidence interval (CI). Variables describing the characteristics and exposure factors associated with *Toxocara* seropositivity in the cases and control subjects were assessed by the Pearson’s *χ*^2^ and Fisher’s exact tests (when expected frequencies were ≤5), in order to identify significant differences between the two groups. The association between *Toxocara* seropositivity and epilepsy was assessed using approximate Bayesian logistic regression employing a penalized likelihood (PL) estimation via data augmentation, and the associated odds ratios (ORs) and 95% CIs were calculated [28]. We introduced a command, “penlogit”, to automatically add specific prior-data records to a data set. These records were computed, so that they generated a penalty function for the log-likelihood of a logistic model, which equals (up to an additive constant) a set of independent log prior distributions on the model parameters [28]. The models were adjusted for potential confounders, including age, gender, and history of epilepsy in parents or relatives; variables were adjusted based on a minimal sufficient adjustment set using directed acyclic graphs (DAGs) [29]. A *p* value of <0.05 was considered as statistically significant.

## 3. Results

### 3.1. Demographic and Clinical Features of Participants

Overall, 182 human subjects (94 epileptic people and 88 healthy controls) were enrolled in the study. Of the 94 patients with epilepsy, 50 (53.2%) were male and 44 (46.8%) were female. The participants’ ages ranged from 13 to 78 years, with a mean age of 36.7 ± 15.9 years. A total of 62 (66%) patients lived in rural and 32 (34%) in urban areas. For 28.7% (27/94) of those with epilepsy, there was a family history of epilepsy, and 22.3% (21/94) had parents with epilepsy. According to the ILAE classification, considering both EEG and clinical data, 28 (29.8%) of the epileptic people presented with focal seizures, while 66 (70.2%) subjects exhibited generalized seizures. Epileptics who were affected by focal and generalized seizures were 37.3 ± 15.1 and 36.8 ± 16.5 years of age, respectively. Controls included 29 males (33%) and 59 females (67%) of 37.5 ± 17.1 years of age. Two control subjects had relatives with a history of epilepsy, and seven had parents with a history of epilepsy. The baseline characteristics of cases and controls are shown in Table 1.

### 3.2. Association between Toxocara Infection/Exposure and Epilepsy

The overall prevalence of anti-*Toxocara* IgG serum antibody in the study subjects was 32.9% (95% CI, 12.2–70.4%; 60/182); 37 of the 94 epileptic patients were positive for anti-*Toxocara* antibody (seroprevalence: 39.3–95% CI, range: 29.4–49.9%); 23 of the 88 control subjects were seropositive for the same antibody (seroprevalence: 26.1–95% CI, range: 17.3–36.5%). In the Pearson’s χ2 test, there was an association between *Toxocara* seropositivity and epilepsy (OR, 1.86; 95% CI, 1.14–3.23; *p* value = 0.019); in a multivariate analysis, when the model was adjusted for covariates, this association was still statistically significant (adjusted OR, 2.38; 95% CI, 1.25–4.63; *p* value = 0.009) (Table 2). 

Of the 28 and 66 epileptic patients with focal and generalized seizures, 12 (42.8%; 95% CI, 24.4–62.8%) and 25 (37.8%; 95% CI, 26.2–50.6%) subjects were seropositive for anti-*Toxocara* IgG serum antibody, respectively. Statistical analyses showed that there was a significant difference in *Toxocara* seropositivity between patients with focal seizures (adjusted OR, 2.60; 95% CI, 1.14–6.04; *p* value = 0.024) or generalized seizures (adjusted OR, 2.17; 95% CI, 1.09–4.40; *p* value = 0.028) and the control group. In addition, seroprevalence was higher in patients with focal seizures than in patients with generalized seizures, although the difference was not significant (adjusted OR, 1.38; 95% CI, 0.63–3.01; *p* value = 0.412) (Table 2). In subgroup analysis, *Toxocara* seropositivity was associated with epilepsy in females (OR, 2.69; 95% CI, 1.14–6.34), PWE aged 40-59 years (OR, 3.9; 95% CI, 1.1–13.8), and those that did not consume unwashed vegetables (OR, 2.67; 95% CI, 1.02–7.01). Additional information is presented in Table 1.

## 4. Discussion

In the present study, we conducted a case–control study to assess the relationship between *Toxocara* spp. infection/exposure and epilepsy in a region in northern Iran. Our findings revealed that incident epileptic patients had a significantly higher seropositivity rate for *Toxocara* (IgG) serum antibody compared with control subjects, suggesting that there is an association between epilepsy and *Toxocara* infection/exposure. These findings are consistent with previous meta-analyses and the hypothesis that *Toxocara* infection/exposure may play a role in triggering the occurrence of epilepsy in people [16,17]. The results are also in accord with some previous studies from the USA [30], Italy [23], Bolivia [31] and Iran [20], showing a significant association, but not with other reports from Turkey [24], Burundi [22], Tanzania [32], India [33] and Egypt [34]. The differences in results between these groups of studies could be due to the type of participants (children, adults or various age groups), the type of epileptic patients (cryptogenic, idiopathic or unspecified), matching criteria and diagnostic methods (ELISA or Western blot analysis). In a relatively recent meta-analysis, Luna et al. (2016) showed a significant association in subgroup analyses, considering the diagnostic method used (Western blot) (OR, 1.79; 95% CI, 1.24–2.59), ‘young’ population (OR, 1.71; 95% CI, 1.02–2.87) and type of study (i.e., population-based) (OR, 1.68; 95% CI, 1.17–2.40), suggesting no variation in findings for different sub-groups [17]. Our findings showed that seropositivity to *Toxocara* was significantly higher in patients with focal seizures than in healthy controls, and also in those with generalized seizures. Few previous studies provided a stratified analysis according to the type of seizures [20,31,35]. Consistent with our findings, studies in Bolivia [31] and Italy [35] indicated a significant association between seropositivity to *Toxocara* and focal seizures, but Zibaei et al. (2013) found an insignificant association between this seropositivity and focal seizures in Iranians [20]. Interestingly, the findings here suggested that females might be more risk of epilepsy; this aspect was not assessed in previous studies and should be critically evaluated in future studies.

Although seroepidemiological studies suggest a relationship between *Toxocara* infection/exposure and epilepsy and other disorders, the pathogenic mechanisms underlying the neurological disorders are not well understood. One proposed mechanism relates to the migration of *Toxocara* larvae to, and through, the CNS [14], which may also account for other neuropsychiatric sequelae related to toxocariasis. Experimental studies in mice have revealed that *Toxocara* larvae can migrate in the tissues of the CNS for up to 28 days and survive for up to 2 years after infection [36]. This migration can result in haemorrhagic lesions, parenchymal damage, focal malacia, demyelination, neuronal necrosis and/or granulomatous alterations in brain [37,38,39,40]. Magnetic resonance imaging (MRI) of the few confirmed epileptic cases apparently linked to *Toxocara* infection revealed granulomatous changes (‘ring-enhancing lesions’) in cortical or sub-cortical regions of the brain [13,41]. Some studies indicated that acute granulomatous reactivity might cause symptomatic seizures, and chronic granulomatous lesions might induce epilepsy [41]. Moreover, migrating *Toxocara* larvae might cause neurodegeneration or neuronal damage, possibly through alterations in neurotransmitter profiles (e.g., gamma-aminobutyric acid, glutamate, dopamine, norepinephrine, and serotonin) [42], elevated expression of nitric oxide synthase (iNOS) and/or pro-inflammatory cytokines (e.g., IL1β, IL6 and TNF-α [43,44]), and/or an increased permeability of the blood-brain barrier [11,42]. It is well established that increased levels of iNOS and pro-inflammatory cytokines can play a role in the development of epilepsy [45,46,47], and that these suggested pathophysiological mechanisms have been proposed also for the epileptogenesis due to neurocysticercosis [48]. Another possible mechanism relates to the autoimmune nature of some types of epilepsy and that *Toxocara* infection can induce the production of autoantibodies [49,50]. There is growing evidence that autoantibodies against neuronal elements might also play a role [51], although the contribution of the autoantibodies produced in the pathogenesis of epilepsy is unclear, and would require detailed investigation [17]. 

## 5. Conclusions

In summary, future efforts are required to elucidate the immunological and molecular mechanisms linked to epilepsy and its association with *Toxocara* and other parasites that can affect the brain and other parts of the CNS. In addition, efforts should focus on prevention and implementing practical programs for the prevention and animal control of *Toxocara*/toxocariasis and other parasitic and zoonotic infections/diseases.

## Figures and Tables

**Table 1:** *Toxocara* seropositivity in epileptic patients and healthy people (control group) according to sociodemographic characteristics subjected to analysis using the Pearson’s chi-square test.

Variable	Patients with Epilepsy [*n* = 94]		Healthy Controls [*n* = 88]		*p* Value	OR_crude_ (95%CI)
	Number (%)	Number Sero- Positive (%)	Number (%)	Number Sero- Positive (%)		
**Sex**						
Male	50 (53.2)	18 (36)	29 (33)	10 (34.48)	0.89	1.07 (0.41–2.79)
Female	44 (46.8)	19 (43.18)	59 (67)	13 (22.41)	0.02 **	2.69 (1.14–6.34)
**Age**						
≤18	13 (13.8)	2 (15.38)	10 (11.4)	3 (30.00)	0.62 *	0.42 (0.06–3.21)
19–39	43 (45.7)	15 (34.88)	45 (51.1)	12 (26.67)	0.40	1.47 (0.59–3.66)
40–59	25 (26.6)	13 (52.00)	23 (27.3)	5 (21.74)	0.03 **	3.90 (1.10–13.80)
≥60	13 (13.8)	7 (53.85)	9 (10.2)	3 (33.33)	0.41 *	2.33 (0.40–13.61)
**Residence**						
Urban	32 (34)	13 (40.63)	32 (36.4)	9 (28.13)	0.29	1.75 (0.62–4.97)
Rural	62 (66)	24 (38.71)	56 (63.6)	14 (25)	0.11	1.89 (0.86–4.18)
**Education**						
Illiterate	12 (12.8)	5 (41.67)	25 (28.4)	7 (28)	0.47 *	1.84 (0.43–7.77)
Primary school	31 (33)	11 (35.48)	27 (30.7)	8 (30.77)	0.63	1.31 (0.43–3.95)
High school	40 (42.6)	15 (37.50)	28 (31.8)	7 (25)	0.28	1.80 (0.62–5.24)
College and above	11 (11.6)	6 (54.55)	8 (9.1)	1 (12.50)	0.14 *	8.40 (0.76–93.34)
**Occupation**						
Farmer	14 (14.9)	7 (50)	31 (35.2)	8 (26.67)	0.17 *	2.88 (0.77–10.77)
House-wife	26 (27.7)	13 (50)	36 (40.9)	9 (25)	0.04	3.00 (1.02–8.81)
Other	54 (57.4)	17 (31.48)	21 (23.9)	6 (28.57)	0.80	1.15 (0.38–3.48)
**Dog contact**						
No	83 (88.3)	32 (38.55)	16 (18.2)	4 (25)	0.30	1.88 (0.56–6.34)
Yes	11 (11.7)	5 (45.45)	72 (81.8)	19 (26.39)	0.28 *	2.32 (0.64, 8.51)
**Cat contact**						
No	81 (86.2)	29 (35.80)	75 (85.2)	20 (26.67)	0.22	1.53 (0.77–3.04)
Yes	13 (13.8)	8 (61.54)	13 (14.8)	3 (23.08)	0.05	5.33 (0.97–29.39)
**Frequent contact** **with soil**						
No	57 (60.6)	22 (38.6)	41 (46.6)	9 (21.95)	0.08	2.23 (0.90–5.56)
Yes	37 (39.4)	15 (40.54)	47 (53.4)	14 (29.79)	0.30	1.61 (0.65–3.98)
**Eating unwashed** **vegetables**						
No	49 (52.1)	20 (40.82)	39 (44.3)	8 (20.51)	0.04 **	2.67 (1.02–7.01)
Yes	45 (47.9)	17 (37.78)	49 (55.7)	15 (30.61)	0.46	1.38 (0.58–3.24)
**Eating raw or undercooked meat**						
No	52 (55.3)	20 (38.46)	52 (59.1)	14 (26.92)	0.21	1.69 (0.74–3.89)
Yes	42 (44.7)	17 (40.48)	36 (40.9)	9 (25)	0.14	2.04 (0.77–5.40)
**Drinking water source**						
Treated	56 (59.6)	25 (44.64)	50 (56.8)	15 (30)	0.12	1.88 (0.84–4.20)
Untreated	38 (40.4)	12 (31.58)	38 (43.2)	8 (21.05)	0.29	1.73 (0.61–4.88)
**Relative with a history of epilepsy**						
Yes	27 (28.7)	11 (40.74)	2 (2.3)	1 (50)	1 *	0.69 (0.04–12.20)
No	67 (71.3)	26 (38.81)	86 (97.7)	22 (25.58)	0.08	1.84 (0.93–3.68)
**Parental history of** **epilepsy**						
Yes	21 (22.3)	8 (38.10)	7 (8)	0 (0)	0.07 *	9.44 (0.48–187.54)
No	73 (77.7)	29 (39.73)	81 (92)	23 (28.40)	0.13	1.66 (0.85–3.26)

* Fisher exact test was used for these variables; ** statistically significant.

**Table 2:** Univariate and multivariate analyses to assess whether there is an association between epilepsy and seropositivity to *Toxocara* in people.

Study Participants	Number Seropositive (%)	Number Seronegative (%)	Univariate Analysis OR (95% CI) *	Multivariate Analysis OR (95% CI) *
Total no. epileptic patients	37 (39.36)	57 (60.64)	1.86 (1.14–3.23)	2.38 (1.25–4.63)
Number of healthy controls	23 (26.44)	64 (73.56)	1	1
Partial epilepsy	12 (41.9)	16 (57.1)	2.04 (1.12–4.21)	2.60 (1.14–6.04)
Healthy controls	23 (26.14)	65 (73.86)	1	1
Generalized epilepsy	25 (37.9)	41 (62.1)	1.79 (1.08–3.21)	2.17 (1.09–4.40)
Healthy controls	23 (26.14)	65 (73.86)	1	1
Partial epilepsy	12 (41.9)	16 (57.1)	1.58 (0.93–3.02)	1.38 (0.63–3.01)
Generalized epilepsy	25 (37.9)	41 (62.1)	1	1

* Crude or adjusted odds ratios (ORs) estimated by approximate Bayesian logistic regression employing a penalized likelihood (PL) estimation via data augmentation [28]; the final multivariable models were adjusted for the following risk factors: age, sex, relatives’ history of epilepsy, and history of epilepsy in a parent.

## Data Availability

The data that support the findings of this study are available from the corresponding author upon reasonable request.
